# A Brief Summary of PET Imaging of Dopaminergic System

**DOI:** 10.1055/s-0045-1802590

**Published:** 2025-01-30

**Authors:** Ismet Sarikaya

**Affiliations:** 1 Department of Nuclear Medicine, Kirklareli University Faculty of Medicine, Kirklareli, Türkiye

Dopamine is one of the major neurotransmitters of the central nervous system involved in various cerebral and peripheral physiological functions and implicated in pathological conditions such as neurologic and psychiatric disorders, cancer, and various benign conditions. 1 There are a large number of positron emission tomography (PET) radiotracers for assessing various elements of the dopaminergic system. Unfortunately, the use of dopaminergic PET tracers in clinical practice is currently limited because the synthesis of most of these tracers requires an on-site cyclotron and radiochemistry expertise, and hence most of them have not yet been approved for clinical use. Among these tracers, only fluorine-18 fluorodopa ( ^18^ F-FDOPA) is commercially available and has been approved for clinical use in cases with suspected parkinsonian syndromes (United States, Europe, and various other places) and certain oncology cases (Europe and some non-European countries). A brief summary of dopaminergic PET tracers and their clinical applications is presented.

Dopaminergic PET radiotracers are classified as pre- and postsynaptic. 2 3 Presynaptic dopaminergic PET radiotracers help assess L-type amino acid and L-DOPA transport via L-amino acid transporters (LATs), dopamine synthesis (DOPA decarboxylase activity) and storage at vesicles (e.g., ^18^ F-FDOPA, ^11^ C-LDOPA, and ^18^ F-fluorotyrosine), dopamine transport via Dopamine transporters (DATs) at presynaptic dopaminergic nerve terminals (e.g., ^11^ C-methylphenidat, ^11^ C-methyl-N-2β-carbomethoxy-3β-(4-fluorophenyl)-tropanel ( ^11^ C-CFT), and ^18^ F-CFT), dopamine transport into vesicles by vesicular monoamine transporter-2 (VMAT-2; e.g., ^11^ C-dihydrotetrabenazine (DTBZ) and ^18^ F-fluoropropyl-DTBZ), and degradation of dopamine by Monoamine oxidase (MAO) enzymes (e.g., ^11^ C-deprenyl, ^11^ C-harmine, ^11^ C-chlorgylline, ^18^ F-fluorodeprenyl, and ^18^ F-rasagaline). Postsynaptic dopaminergic PET radiotracers mainly assess postsynaptic dopamine receptors (e.g., ^11^ C-(R)-(+)-8-chloro-2,3,4,5-tetrahydro-3-methyl-5-phenyl-1H-3- benzazepin-7-ol) ( ^11^ C-SCH 23390) that bind to D1 receptors, and ^11^ C-raclopride, ^18^ F-fallypride, ^11^ C-methylspiperone, and ^18^ F-desmethoxyfallypride that bind to D2 and D3 receptors). ^18^ F-fluorodopamine is a radiolabeled dopamine analog that reflects dopamine transfer and storage in peripheral catecholamine-synthesizing cells.

In the brain, dopaminergic PET tracers have wide applications including parkinsonian syndromes, Huntington's disease, dementias, schizophrenia, drug addictions, and various other conditions. 3 In patients with parkinsonian syndromes, presynaptic dopaminergic PET tracers help differentiate neurodegenerative parkinsonism from essential tremor and non-neurodegenerative parkinsonism (e.g., drug-induced, vascular, and psychogenic parkinsonism). 4 Because of upregulation of DOPA decarboxylase and downregulation of DAT in presymptomatic phase of Parkinson's disease (PD), DAT PET may be more sensitive than ^18^ F-FDOPA for earlier detection of PD. Typical findings include progressive decline in radiotracer uptake in the striatum, contralateral to the most clinically affected side. In advanced disease, both striata are involved. Initially reduced uptake is located in the posterior putamen, but with the progression of the disease, it extends to the anterior putamen and finally to the caudate nucleus. Postsynaptic dopamine receptor PET imaging helps differentiate PD from other atypical neurodegenerative parkinsonian syndromes (multiple system atrophy, corticobasal degeneration, and progressive supranuclear palsy) as they have neurodegeneration both at pre- and postsynaptic levels. 5 In the cases with dementia, dopaminergic PET imaging with ^18^ F-FDOPA and DAT tracers help differentiate dementia with Lewy bodies (DLB) from Alzheimer's disease (AD) with reduced uptake in the striatum in most DLB cases. 6 PET tracers assessing MAO enzymes help assess neuroinflammation and astrocytosis in AD. In Huntington's disease, PET studies have showed reduced striatal binding of various dopaminergic radiotracers targeting D1 and D2 receptors and DAT. 7 In psychiatry, various dopaminergic PET tracers have been widely studied in schizophrenia, drug addictions, and other psychiatric conditions. In patients with schizophrenia, significantly higher levels of dopamine synthesis capacity in the striatum and reduced binding to D1 receptors in the prefrontal cortex were some of the reported results of PET studies. 3

In oncology, dopaminergic PET imaging covers a variety of cancers (e.g., paragangliomas, pheochromocytoma, medullary thyroid carcinoma, gut carcinoids, and gliomas) as there is overexpression of LAT-1 and also DOPA decarboxylase and certain dopamine receptors in various cancers. 3 8 ^18^ F-FDOPA PET imaging has high sensitivity in detecting pheochromocytomas (benign or metastatic) and paragangliomas of the head and neck (parasympathetic origin). 9 ^18^ F-FDOPA PET is particularly more sensitive in detecting metastases of succinate dehydrogenase B (SDHB)-negative cases than SDHB-positive cases of paragangliomas and pheochromocytoma. Studies have demonstrated that ^18^ F-FDOPA PET is more sensitive than ^123^ I-metaiodobenzylguanidine ( ^123^ I-MIBG) scan in detecting neuroblastoma lesions (staging and relapse) and evaluating disease persistence after chemotherapy (prognostic value). 10 ^18^ F-FDOPA PET is also useful for initial diagnosis and grading (differentiating low from high grade) of newly diagnosed gliomas, detecting recurrences, assessing response to treatments, differentiating recurrence from radiation necrosis and pseudo-progression, and determining the extent of primary and recurrent tumor for treatment planning. 11 In medullary thyroid carcinoma, ^18^ F-FDOPA PET is useful in detecting recurrence and its extent in cases with rising tumor markers, with increasing detection rate at higher levels and shorter doubling time of serum calcitonin. 12 In neuroendocrine tumors (NETs), ^18^ F-FDOPA PET is useful in detecting the primary tumor in suspected cases and detecting recurrences in well-differentiated and low-grade NETs or carcinoids of midgut (distal jejunum, ileum, appendix, and right colon). 13

In cases with hyperinsulinemic hypoglycemia, ^18^ F-FDOPA PET is considered the radiotracer of choice for the detection and localization of focal form of congenital hyperinsulinemia of infants, which allows surgical resection of a focal lesion. 14 ^18^ F-FDOPA PET with carbidopa premedication and dual time point acquisition is a valuable imaging technique to localize insulinoma in adult patients. 15
